# Intermetallic Layers with Tuned Na Nucleation and Transport for Anode-Free Sodium Metal Batteries

**DOI:** 10.1021/acs.nanolett.4c04282

**Published:** 2025-01-27

**Authors:** Jie Shi, Danni Wang, Qun Liu, Zhenlu Yu, Jian-Qiu Huang, Biao Zhang

**Affiliations:** † Department of Applied Physics and Research Institute for Advanced Manufacturing, The Hong Kong Polytechnic University, Hung Hom, Hong Kong 999077, China; ‡ Department of Physics and Energy, Chongqing Key Laboratory of New Energy Storage Materials and Devices, Chongqing University of Technology, Chongqing, 400054, China

**Keywords:** anode-free batteries, thin interphase layer, sodiophilic, sodium metal anodes

## Abstract

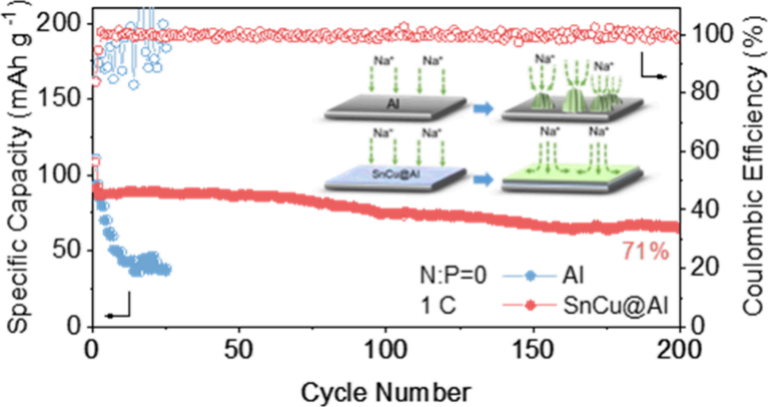

Sodium metal batteries without pre-deposited Na (anode-free) and with a limited amount of Na metal (anode-less) have attracted increasing attention due to their competitive energy density and the high abundance of sodium. However, severe interfacial issues result in poor cycling stability and low Coulombic efficiency. Here, the lightweight interphase layers composed of intermetallic nanoparticles (Sn–Cu and Sn–Ni) are applied to improve Na plating/stripping behaviors. These layers provide uniform seeding sites with high sodiophilicity and support fast ion transport. A reversible Na plating/stripping behavior, featuring a high Coulombic efficiency of ∼99.95% with a minor standard deviation of 0.0013, for 500 cycles at 1 mA cm^–2^ and 1 mAh cm^–2^ is achieved on SnCu-coated Al. Consequently, the anode-free Na_3_V_2_(PO_4_)_3_ full cell with a high loading of 7.6 mg cm^–2^ exhibits a capacity retention of 90% after 200 cycles. This strategy provides an effective pathway toward anode-free sodium metal batteries.

With the growing demand for electric vehicles and stationary energy storage, the development of rechargeable batteries with low cost and high energy density is in urgent need.^1,2^ Rechargeable sodium metal batteries (NMBs) stand out as a viable supplement to lithium batteries in energy storage systems, owing to the high natural abundance of Na and high theoretical capacity (1166 mAh g^–1^) of the Na anode.^3^ Besides, the high Na reversibility on Cu with Coulombic efficiency (CE) of 99.9% at 0.5 mA cm^–2^ in the glyme-based electrolyte makes it appealing for practical long-term cycling.^4^ However, the energy density of NMBs remains unsatisfactory, as a great excess of Na metal is usually required.^5^ Pairing the Na-containing intercalation cathode with a bare current collector gives the so-called anode-free battery significantly improved energy density. Besides, without thermodynamically reacting with Na ions, the lightweight Al can be applied as a current collector on both the cathode and anode sides.^3^ It holds significant potential for enabling cost-effective battery manufacturing. Nevertheless, the cyclic lifespan of an anode-free battery is largely dependent on the reversibility of Na plating/stripping due to the lack of Na inventory. The poor Na affinity of Al causes an inhomogeneous Na ion distribution, leading to the preferential formation of dendrites.^6,7^ During the repeated cycling, the nonuniform grown Na would cause the breakage and reformation of a solid electrolyte interphase (SEI), resulting in reduced CE and depletion of active Na.^8,9^ Consequently, anode-free batteries usually show rapid capacity decay.

Many promising strategies have been investigated to improve the cycling stability of anode-free NMBs, including electrolyte design,^10−15^ artificial SEI construction,^16,17^ and current collector modification.^7,18−21^ Among them, modification of the current collector is highly effective, as it significantly influences the initial Na nucleation and the subsequent plating. To date, various avenues have been explored, such as 3D porous hosts,^18,22^ carbon coating,^19^ and producing sodiophilic metal layers,^23−25^ to reduce the nucleation overpotential and guide uniform Na plating. Among these, Sn coating is widely studied for its ability to reduce the nucleation barrier and promoting uniform Na^+^ ion transport by forming a sodiophilic Sn–Na alloy.^26−29^ The introduction of Sn can provide nucleation seeds for Na and promote uniform deposition.^8,28^ However, in many cases, the improved electrochemical performance is accompanied by additional volume and mass, sacrificing the energy densities at the cell level. Moreover, the Sn layer would undergo pulverization and gradually detach from the current collector after repeated cycling, resulting in limited cycle life.^30^

Herein, we report a thin interphase layer (∼20 nm) composed of intermetallic compounds (SnNi and SnCu) on bare Al to enable highly reversible Na plating/stripping behaviors. The intermetallic compounds (Sn–M) are constructed through pairing up the counterpart metals (M) with the active Sn. Cu and Ni, chosen as counterparts, provide high conductivity and act as mechanical buffers to strengthen the structure and alleviate the disintegration of the Sn.^31,32^ The Sn–Ni and Sn–Cu alloys are simultaneously formed through the co-sputtering of two metal targets. The lightweight coating would impose a negligible influence on the energy density of the whole battery system. The uniformly distributed SnNi and SnCu nanoparticles provide abundant seeding sites and promote Na ion diffusion. With dendrite-free and dense Na plating, the consumption of active Na is effectively suppressed. Therefore, high Na plating/stripping stability with low interfacial resistance is realized. The SnCu-coated Al foils exhibit a high CE of ∼99.95% and a small standard deviation of 0.0013, for 500 cycles at 1 mA cm^–2^ and 1 mAh cm^–2^. Benefiting from such an interphase layer, the anode-free Na_3_V_2_(PO_4_)_3_ (NVP) full cell with a high loading of 7.6 mg cm^–2^ shows improved cycling stability with a capacity retention of 90% after 200 cycles at 1 C. Our strategy offers a promising solution to enhance the functional coating layer and provides new insights into the development of high-energy anode-free NMBs with an intermetallic SnNi and SnCu coating.

Magnetron co-sputtering is involved in constructing a thin layer with nanosized particles. The layers of Sn, SnNi, and SnCu on bare Al were denoted as Sn@Al, SnNi@Al, and SnCu@Al, respectively. Figure 1a shows a schematic illustration of the Na plating process. The intermetallic compounds on the Al surface are expected to generate a Na–Sn alloy. With the low diffusion barrier, the Na–Sn alloy would serve as efficient sodiophilic seeding sites and promote the epitaxial growth of Na along the interphase layer.^33,34^ In the meantime, Cu/Ni metal can provide structural support for the Na–Sn alloy. Therefore, the smooth and dendrite-free Na plating can be realized and maintained during cycling, significantly outperforming the bare Al.^35^

**Figure 1:**
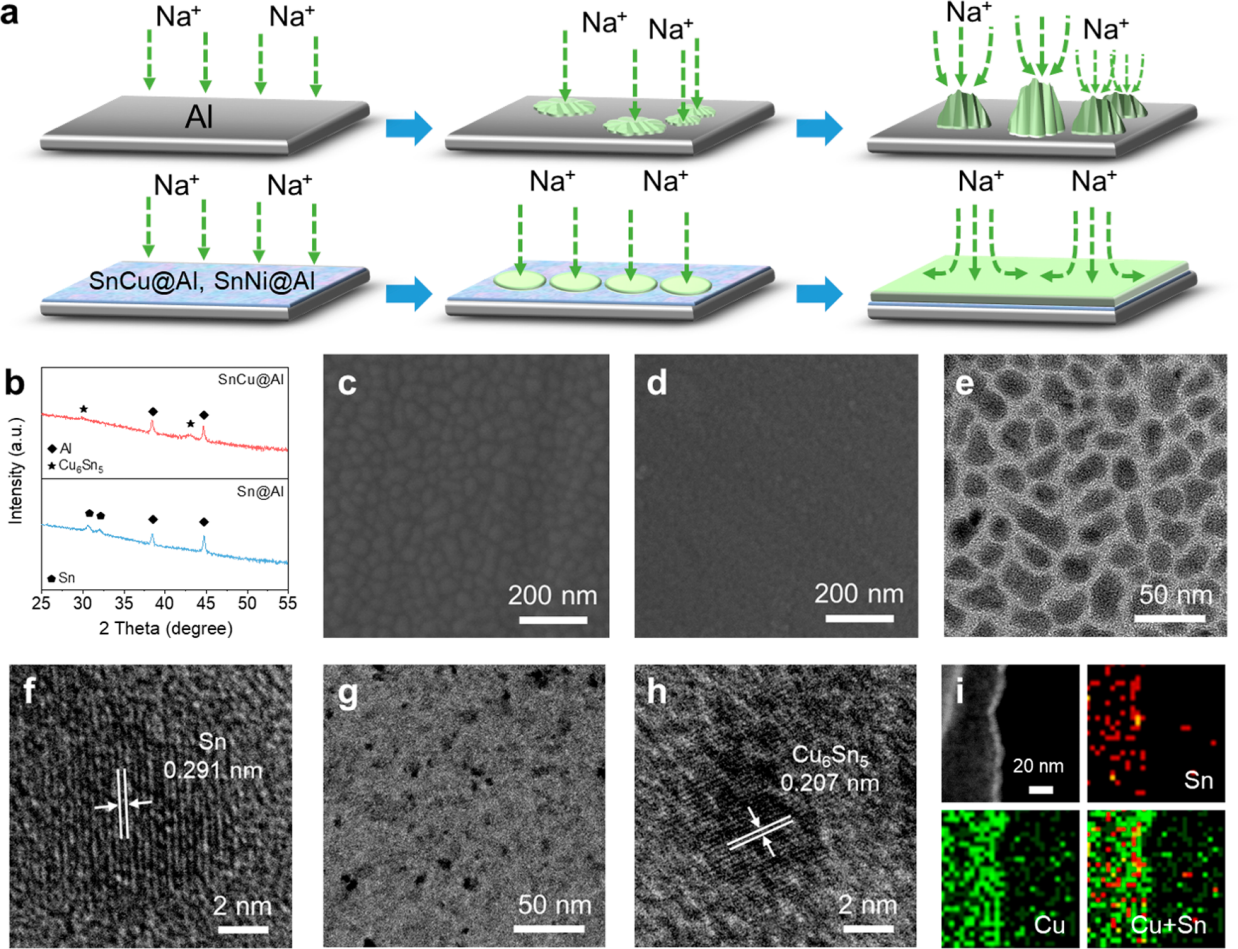
Preparation of the intermetallic coating layer. (a) Schematic illustration of Na plating on Al, SnNi@Al, and SnCu@Al. (b) XRD patterns of Sn@Al and SnCu@Al. SEM images of (c) Sn@Al and (d) SnCu@Al. (e) TEM and (f) HRTEM images of the Sn interphase layer. (g) TEM and (h) HRTEM images of the Sn–Cu interphase layer. (i) EDS elemental mapping of the Sn–Cu interphase layer.

The Al foils (Figure S1) show the transition of color from bright gray to dark gray after sputtering, indicating the full coverage after sputtering. The ultralow mass loading (<0.03 mg cm^–2^) and nanoscale thickness (∼20 nm) would hardly impose a sacrifice in the energy density of the whole battery configuration (Figure S2). The composition of the layers was verified by X-ray diffraction (XRD) characterization, as shown in Figure 1b and Figure S3. Two identified peaks at 38.5° and 44.7° are attributed to baseline Al (JCPDS No. 89-4037). In the case of Sn@Al, two peaks at 30.7° and 32.0° belong to metallic Sn (JCPDS No. 65-2631). Peaks at 30.4° in SnNi@Al and 29.9° and 42.9° in SnCu@Al prove the formation of Ni_3_Sn_2_ (JCPDS No. 06-0414) and Cu_6_Sn_5_ (JCPDS No. 47-1575), respectively. A scanning electron microscope (SEM) was utilized to investigate the surface morphology of the as-made specimens. Figure 1c illustrates that Sn@Al exhibits a distinct surface texture compared with the Al foil (Figure S4), featuring uniformly distributed particles ranging from 30 to 50 nm in diameter. As for SnNi@Al, the coating exhibits a nonuniform distribution of both smaller and larger particles (Figure S5). With the co-sputtering of Sn and Cu, the surface of SnCu@Al is flatter, with smaller particle sizes and increased interconnection between nanoparticles (Figure 1d). The variations in grain sizes could be attributed to the generation of stress resulting from the disparity in atom sizes between Sn and counterpart metals.^36^ The smaller particle sizes on the layer would prevent the Na aggregation into large clusters and exhibit enhanced stability during the repeated plating/stripping process.^37^ The morphological characteristics of Sn and SnCu layers were further confirmed by transmission electron microscopy (TEM) through direct sputtering on a Cu mesh. As shown in Figure 1e, the grain size and the distribution of Sn nanoparticles are in agreement with SEM results. With the addition of Cu, the SnCu nanoparticles appear to be smaller than single Sn (Figure 1g). From high-resolution TEM (HRTEM) images (Figure 1f,h), lattice fringes with spacings of 0.291 and 0.207 nm correspond to the Sn (200) and Cu_6_Sn_5_ (102) planes, respectively. Elemental mapping imaging (Figure 1i) by energy-dispersive X-ray spectroscopy (EDS) reveals the even distribution of Sn and Cu elements.

To verify the effect of SnCu@Al on Na plating/stripping behaviors, asymmetric Al/Na cells with various substrates were tested under 1 M sodium hexafluorophosphate (NaPF_6_) in a diglyme electrolyte. Figure S6 shows the galvanostatic profile of the SnCu@Al employed for Na activation at 0.1 mA cm^–2^ with a cutoff voltage of 1 V. During the activation process, a conversion reaction takes place wherein Cu–Sn reacts with Na to generate Na–Sn and Cu. The sodiation process is characterized by a single plateau located around 0.05 V, whereas two plateaus at approximately 0.2 and 0.6 V emerge during the desodiation process.^38^ To evaluate the sodiophilicity of different substrates, the initial Na plating process was measured at 0.2 mA cm^–2^. With interphase layers, the Na–Sn alloying plateau can be observed during the first discharge (Figure 2a and Figure S7). Benefiting from the favorable Na affinity of the Na–Sn alloy, the nucleation overpotentials on Sn@Al, SnNi@Al, and SnCu@Al are 7, 6, and 6 mV, respectively, significantly lower than bare Al (82 mV). The results suggest a lower nucleation barrier on the substrate enhanced with intermetallic compounds. XRD was further carried out to investigate the changes in the structure and composition evolution. A certain amount of Na (0.01 and 0.05 mAh cm^–2^) was plated on the SnCu@Al electrode at a small current density of 0.05 mA. As shown in Figure S8, the peaks for the Cu–Sn alloy can be observed within the initial plating state (0.01 mAh cm^–2^) and the thicker Na plating state (0.05 mAh cm^–2^). The XRD pattern at 0.05 mAh cm^–2^ evidences the formation of Na_15_Sn_4_, implying the formation of a Na–Sn alloy. Based on the capacity of the Na–Sn alloy (∼0.0107 mAh) and the mass of the coating layer, it could be inferred that around 65% SnCu participated in the conversion and alloy reactions. The coexistence of Na_15_Sn_4_ and Cu–Sn alloy proves that the Cu–Sn interface layer is not fully reacting with Na. As a result, the restrained amount of the Na–Sn alloy would induce less volume expansion and alleviate the disintegration of the Sn. The electrochemical reversibility on different substrates was further evaluated. A high average CE of 99.84% during 500 cycles is observed for bare Al at 1 mA cm^–2^ and 1 mAh cm^–2^ (Figure 2b). Nevertheless, it exhibits a large standard deviation (Std. Dev.) of 0.0081 (0.81%), suggesting fluctuating CE values. CE values over 100% on some cycles on bare Al indicate the presence of excessive “dead” Na that could occasionally reconnect.^39^ Hence, when evaluating the Na plating/stripping behavior with CE over 99%, it is important to consider not only the average CE but also the Std. Dev. as a crucial factor. Here, after the effective coating on Al, Sn@Al exhibits improved cycling stability with a Std. Dev. of 0.0028 (Figure 2b). As for SnNi@Al and SnCu@Al, the stable Na plating/stripping behavior with a higher average CE of 99.93% and 99.95% and lower Std. Dev. of 0.0023 and 0.0013 can be observed, respectively (Figure 2b and Figure S9). The better electrochemical performance of SnCu@Al than SnNi@Al can be deduced from the smaller grain size and better coverage of the Al substrate; hence we next focus on SnCu@Al for exploring the underlying mechanism. Under harsher conditions, i.e., 3 mA cm^–2^ and 3 mAh cm^–2^, SnCu@Al maintains a high average CE of 99.98% during 500 cycles, whereas the cell with bare Al fails drastically after only 190 cycles (Figure 2c). The detailed voltage profiles during the 451–455 cycles (Figure 2d) prove a more stable stripping process with low voltage hysteresis on SnCu@Al. These results suggest that SnCu@Al can facilitate homogeneous Na plating and improve electrochemical reversibility.

**Figure 2:**
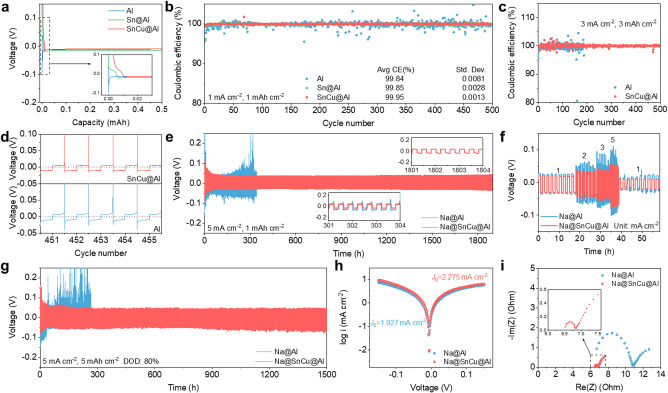
Na plating/stripping stability. (a) Nucleation overpotentials of Na plating on different substrates at 0.2 mA cm^–2^. CE of Na/Al half cells at (b) 1 mA cm^–2^ and 1 mAh cm^–2^ and (c) 3 mA cm^–2^ and 3 mAh cm^–2^. (d) Voltage profiles of Na/Al half cells from 451 to 455 cycles at 1 mA cm^–2^ and 1 mAh cm^–2^. (e) Cycling performance of Na/Na symmetrical cells using Na@Al and Na@SnCu@Al electrodes at 5 mA cm^–2^ and 1 mAh cm^–2^. (f) Rate performance at different current densities. (g) Cycling performance at 5 mA cm^–2^ and 5 mAh cm^–2^ with 80% DOD. (h) Tafel plots and (i) Nyquist plots of Na/Na symmetrical cells after 100 cycles at 1 mA cm^–2^ and 1 mAh cm^–2^.

The symmetric cells were fabricated with a certain amount of Na (6.25 mAh cm^–2^) predeposited on the substrate to further evaluate the stability and reversibility of the Na@SnCu@Al electrode. As shown in Figure 2e, the cell with Na@Al exhibits unstable voltage hysteresis and fluctuations only after 100 h at 5 mA cm^–2^ and 1 mAh cm^–2^ (∼16% depth of charge, DOD). By contrast, the Na@SnCu@Al electrode demonstrates an extended cycle life of 1800 h with a low overpotential of ∼40 mV. This observation highlights the significance of the SnCu intermetallic compound in stabilizing the plating/stripping process and promoting fast reaction kinetics. The rate performances of symmetric cells are shown in Figure 2f and Figure S10. At current densities from 1 to 5 mA cm^–2^ with a fixed capacity of 1 mAh cm^–2^, the Na@SnCu@Al electrode delivers a lower overpotential at each current density than Na@Al. As shown in Figure S10, the symmetric cells were further tested at 1–10 mA cm^–2^ with a capacity of 1 mAh cm^–2^. The Na@SnCu@Al symmetric cell shows considerably lower voltage hysteresis at each current density. At a high current density of 10 mA cm^–2^, the voltage hysteresis of the Na@SnCu@Al symmetric cell (∼260 mV) is lower than the bare Na (∼400 mV) and Na@Sn@Al (∼280 mV). Apart from the current density and areal capacity, Na plating/stripping stability can also be influenced by the Na utilization.^40^ Under a harsh condition of 5 mA cm^–2^ and 5 mAh cm^–2^ with 80% DOD, the Na@SnCu@Al electrode remains stable for 1500 h (Figure 2g). To evaluate the charge transfer kinetics of Na plating/stripping at interfaces, Tafel plots were generated by collecting linear sweep voltammetry (LSV) data to investigate the exchange current density (*J*_0_).^41^ As shown in Figure 2h, the *J*_0_ value for Na@SnCu@Al is 2.275 mA cm^–2^, higher than the corresponding value of Na@Al (1.927 mA cm^–2^). This indicates that Na@SnCu@Al exhibits a faster charge transfer than Na@Al. Electrochemical impedance spectroscopy (EIS) was further conducted on symmetric cells after 200 cycles at 1 mA cm^–2^ and 1 mAh cm^–2^ (Figure 2i). The EIS curves are analyzed by fitting with an equivalent circuit model, as shown in Figure S11. Na@SnCu@Al shows a lower interfacial resistance than Na@Al, reinforcing the results from Tafel plots that the SnCu@Al electrode enables a favorable Na ion charge transfer.

The effect of SnCu@Al on the evolution of Na plating morphology was further investigated by SEM with different areal capacities from 0.2 mAh cm^–2^ to 1 mAh cm^–2^ (Figure 3a). Within the initial plating process, the Al is covered incompletely with nonuniform Na metal, reflected by a surface of uneven brightness. As the plating capacity increases to 1 mAh cm^–2^, the Na on Al grows into an irregular and dendritic structure with randomly oriented filaments. In contrast, the SnCu@Al electrode is fully covered at the initial stage, and the smooth surface is maintained throughout the plating process. Optical images with different plating capacities were also obtained to compare the surface appearance (Figure S12). Island-like Na particles appear randomly on the bare Al with 0.2 mAh cm^–2^ Na plating. With an increased capacity, these particles gradually merge and partially cover the surface. In the case of SnCu@Al, smaller Na islands can be found. The compact Na plating exhibits a uniform orientation and full coverage on the substrate. From above, it can be deduced that with low-barrier seeding sites, the uniformly oriented SnCu nanoparticles can guide the low-porosity growth, thus leading to uniform Na plating. Atomic force microscopy (AFM) was further employed to characterize the morphology of the plated Na. Figure 3b shows the 3D topographic images of Al and SnCu@Al with different areal capacities, with brightness signaling the height of the position. Ra represents the surface roughness, which is calculated by measuring the deviation of surface height from the average plane. The Al electrode shows the largest Ra values of all the Na plating capacities, demonstrating the poor sodiophilic and heterogeneous surface. By contrast, the smoother surface of SnCu@Al suggests its benefit in regulating the Na plating process. Besides, to check the stability of the intermetallic surface layer after long cycling, the surfaces of Sn@Al and SnCu@Al electrodes after being fully stripped of Na were detected by AFM. Figure S13 shows the surface of Sn@Al and SnCu@Al after 100 cycles of plating and stripping at 2 mA cm^–2^ and 1 mAh cm^–2^. Obvious aggregation can be observed on the Sn@Al electrode, and the observable lines indicate the exposed Al surface. The SnCu@Al electrode show a rather smooth surface without exposure of Al. These results confirm the better stability of SnCu@Al in long-term cycling.

**Figure 3:**
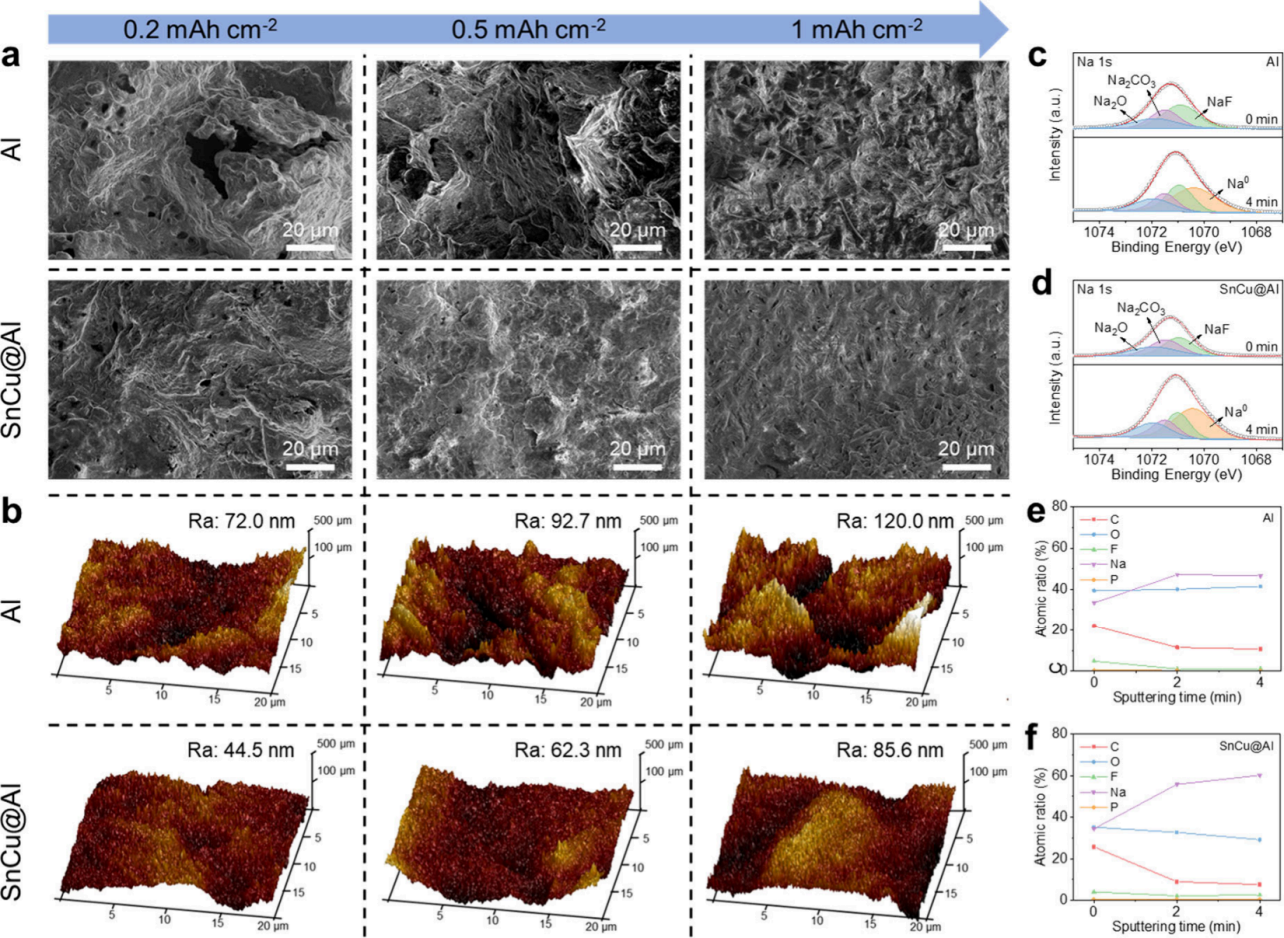
Na deposition behavior. (a) SEM and (b) AFM 3D topography images of Na plating on bare Al and SnCu@Al with a capacity of 0.2, 0.5, and 1 mAh cm^–2^ at 0.5 mA cm^–2^, respectively. In-depth Na 1s XPS spectrum of Na plating on (c) Al and (d) SnCu@Al electrodes after 30 cycles at 2 mA cm^–2^ and 2 mAh cm^–2^. Corresponding depth profiles of the atomic ratio of C, O, F, Na, and P elements on (e) Al and (f) SnCu@Al electrodes.

We also collected X-ray photoelectron spectroscopy (XPS) to examine the effect of coating on the chemical composition. The electrodes were cycled for 30 cycles at 2 mA cm^–2^ and 2 mAh cm^–2^ to form the stable interphases (Figure 3c,d and Figure S14). At the outer SEI structure (without Ar^+^ etching), both Al and SnCu@Al electrodes show intensive peaks of C–C (284.8 eV), C–O (286.7 eV), and O–C=O (corresponding to Na_2_CO_3_) (289.2 eV) in C 1s spectra, correlating with the organic species (sodium alkoxides) and inorganic species (sodium carbonate).^4,14,42^ The intensity of the peak for O–C=O is larger than that of C–C and C–O. The carbon contents of SEI obtained from SnCu@Al electrodes after sputtering show smaller intensities than those on the Al foil. The negligible signal of C–O indicates less decomposition during the repeated Na plating/stripping process. The intensive peaks at 1070.9, 1071.5, and 1071.9 eV in Na 1s spectra are consistent with NaF, Na_2_CO_3_, and Na_2_O.^43,44^ After 4 min of Ar^+^ etching, the intensities of organic signals (C–C, C–O) attenuate, while the inorganic NaF, Na_2_CO_3_, and Na_2_O signals on the Al electrode are stable. The peak at 1070.5 eV corresponding to Na^0^ suggests the penetration of the SEI.^10^ The SnCu@Al electrode shows reduced carbon-containing peaks and an enhanced Na^0^ peak, suggesting a thinner SEI. According to the element distribution in Figure 3e, the remaining high content of C (10.6 at. %) after 4 min of Ar^+^ etching on the Al electrode indicates the great number of organic decompositions from the inner layer with high resistance.^42^ On the SnCu@Al electrode, the C content significantly decreases from 25.8 to 7.5 at. %. The Na content features a constant growth to 60.2 at. % after 4 min of etching, suggesting a thinner SEI layer that can facilitate fast ion transport (Figure 3f).^45^ These findings prove that the regulated Na plating process can be achieved with this SnCu intermetallic compound, aligning with the results of Tafel plots and EIS curves.

To further evaluate the feasibility of the SnCu@Al electrode in practical applications, anode-less and anode-free full NVP battery configurations were fabricated. With a capacity ratio of negative to positive (N/P ratio) of 1, Na@Al/NVP and Na@ SnCu@Al/NVP cells were tested at 1 C (1 C = 117 mAh g^–1^). As shown in Figure 4a, the Na@Al/NVP cell exhibits inferior cycling stability with a rapid failure within 50 cycles. This could be caused by repeated side reactions and the continuous consumption of active Na. The sharp capacity decay can also be observed from the charge/discharge curves, as shown in Figure S15a. The Na@SnCu@Al/NVP cell shows a long lifespan of 400 cycles with a high capacity retention of 93%, demonstrating the crucial role of SnCu in stabilizing NMBs. Furthermore, the anode-free cells were examined with Al, Sn@Al, and SnCu@Al without pretreatment. As shown in Figure 4b, due to the lack of a Na supplement on the anode side, an initial discharge capacity of 93.8 mAh g^–1^ at 1 C is delivered in the Al/NVP cell. The fluctuating CE values can be noticed, and the discharge capacity fails drastically, to 52.5 mAh g^–1^ within 20 cycles. Anode-free Sn@Al/NVP cells show a stable performance during the initial 50 cycles but a gradual capacity decay with cycling (Figure S16). By contrast, the SnCu@Al/NVP cell displays a reversible capacity of 64.5 mAh g^–1^ and a capacity retention of 71% after 200 cycles. Figure 4c shows the voltage profiles of Al/NVP and SnCu@Al/NVP in different cycles. The unsmooth profiles after 5 cycles can be attributed to the unstable interface between the anode and electrolyte in the Al/NVP cell. In comparison, the SnCu@Al/NVP cell delivers flatter profiles with lower voltage hysteresis. Besides, the SnCu@Al/NVP cell was tested at 2 C. The SnCu@Al/NVP cell shows an impressive capacity retention of 82% after 100 cycles, while the Al/NVP cell shows a sharp capacity loss and decays within 20 cycles. Figure 4e and Figure S17 show the rate capability of Sn@Al/NVP, and SnCu@Al/NVP cells with current densities increasing from 1, 2, 4, 6, and 10 C. The SnCu@Al/NVP cell exhibits significantly improved capacities under every current density compared to the Sn@Al/NVP cell. The SnCu@Al/NVP cell shows reversible capacities of 86.5, 84.7, 79.5, 73.9, and 67.2 mAh g^–1^, respectively. Upon returning from 10 C to 2 C, a specific capacity of 76 mAh g^–1^ can be achieved. At a current density of 585 mA g_NVP_^–1^ (5 C), the anode-free cell utilizing SnCu@Al demonstrates remarkable stability throughout 100 cycles, retaining 79% of its initial capacity (Figure S18). Furthermore, the full cell is tested with stricter conditions of a high loading of cathode (7.6 mg cm^–2^). As shown in Figure 4f, the cell could maintain stable cycling for 200 cycles with a high average CE value (99.79%) and high capacity retention (90%), indicating the superb stability of the SnCu interphase layer. The findings confirm that SnCu@Al greatly boosts the performance of anode-free NVP full cells.

**Figure 4:**
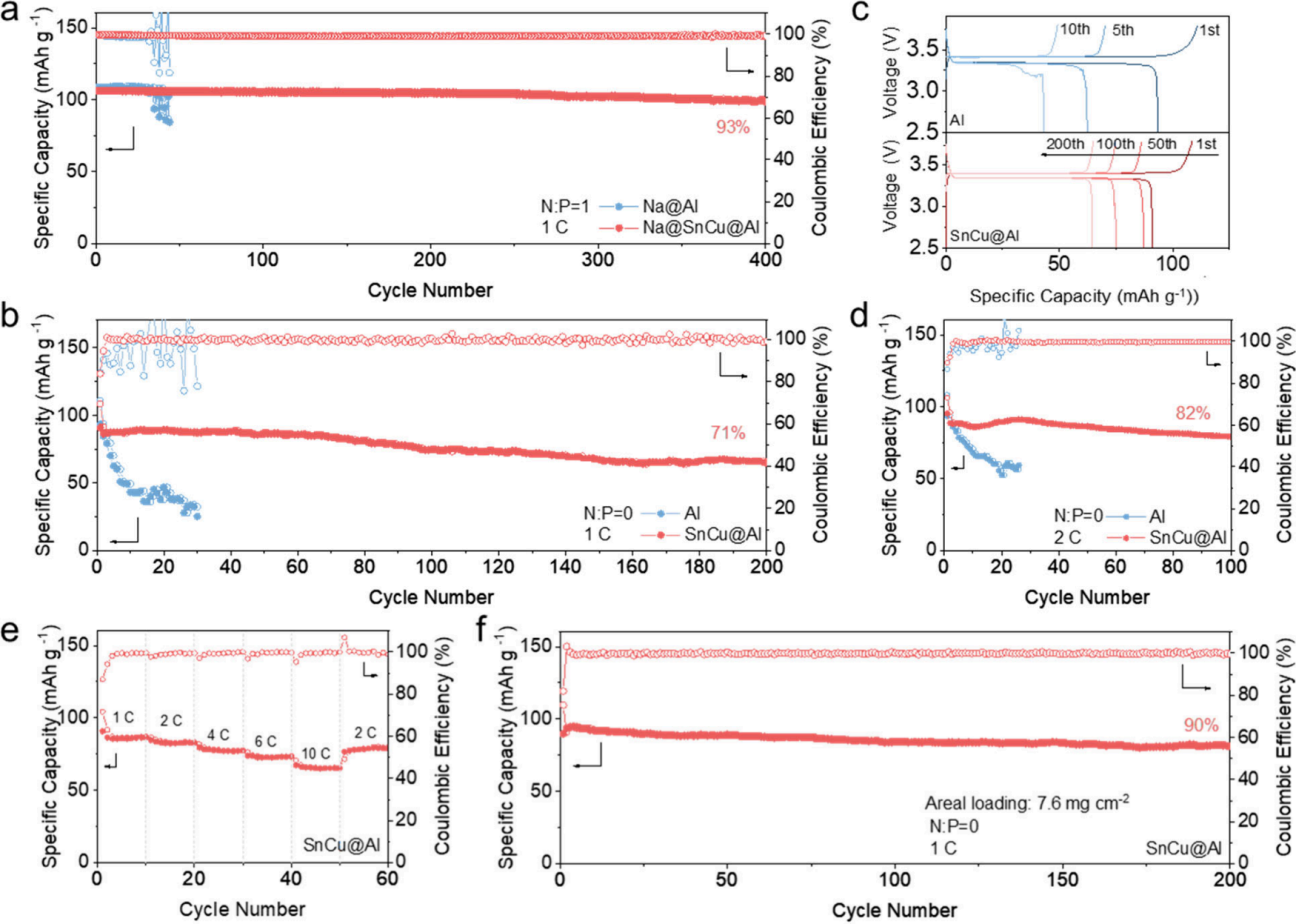
Full cell evaluation. (a) Cycling performance of Na@Al/NVP and Na@SnCu@Al/NVP full cells at 1 C. (b) Cycling performance of Al/NVP and SnCu@Al/NVP anode-free full cells at 1 C. (c) Corresponding charge/discharge profiles of Al/NVP and SnCu@Al/NVP at different cycles. (d) Cycling performance of Al/NVP and SnCu@Al/NVP full cells at 2 C. (e) Rate performance of the SnCu@Al/NVP cell cycling at increasing rates from 1 C to 10 C. (f) Cycling performance of the SnCu@Al/NVP cell with a high areal loading of 7.6 mg cm^–2^ at 1 C.

In summary, we have developed thin interphase layers with intermetallic compounds to provide uniform Na plating and restrict dendrite growth. The nanosized SnCu and SnNi particles can provide heterogeneous seeds for Na nucleation and promote further Na diffusion in the current collector. Benefiting from the modified electrodes, highly reversible Na plating/stripping behaviors and long-term cycling stability are exhibited in both half cells and symmetric cells. The SnCu@Al electrode delivers a high average CE of ∼99.95% for 500 cycles with a small Std. Dev. of 0.0013 at 1 mA cm^–2^ and 1 mAh cm^–2^. The symmetric cells exhibit a high Na utilization with 80% DOD at 5 mA cm^–2^ and 5 mAh cm^–2^. The SnCu@Al electrode is also successfully applied in anode-less and anode-free NVP full cells. An impressive capacity retention of 90% over 200 cycles at 1 C in the SnCu@Al/NVP cell with a high loading of 7.6 mg cm^–2^ is achieved. This study provides a feasible pathway toward high-energy anode-free NMBs with a facile design of the current collector.
