# Supplementary material for: Intermetallic Layers with Tuned Na Nucleation and Transport for Anode-Free Sodium Metal Batteries
Source: Nano Lett. 2025 Jan 27;25(5):1800–7. doi: 10.1021/acs.nanolett.4c04282 (PMC11803737; doi:10.1021/acs.nanolett.4c04282)
Supplement: Supplementary file 1 — nl4c04282_si_001.pdf [file nl4c04282_si_001.pdf]

Supporting Information

Intermetallic layers with tuned Na nucleation and transport for Anode-Free Sodium Metal Batteries

Jie Shi[a], *Danni Wang*[a], *Qun Liu*[a], *Zhenlu Yu*[a], *Jian-Qiu Huang*[b, *], *Biao Zhang*[a, *]

[a]Department of Applied Physics and Research Institute for Advanced Manufacturing, The Hong Kong Polytechnic University, Hung Hom, Hong Kong, 999077, China

[b]Department of Physics and Energy, Chongqing Key Laboratory of New Energy Storage Materials and Devices, Chongqing University of Technology, Chongqing, 400054, China

Experimental Section/Methods

Materials: Sodium metal (99.9%) was purchased from Sigma. Aluminum foil (18 μm, 99.35%) and $Na_3V_2(PO_4)_3$ (NVP) powder were purchased from Kejing Star Technology Corp. The electrolyte was prepared by dissolving 1 M (mol L^{-1}) $NaPF_6$ (Duoduo chem, 99%) in diglyme (Sigma, 99.5%) inside an argon-filled glovebox.

Preparation of Sn@Al, SnNi@Al, and SnCu@Al: Al foil was first cut into one 10 cm x 10 cm piece and washed with ethanol. The Sn@Al, SnNi@Al, and SnCu@Al electrodes were prepared with magnetron sputtering. The distance between the target and Al foil was 10 cm. The power of generators for Cu target, Nickel and Sn target were adjusted to 40 W, 150 W and 20 W, respectively. The sputtering time was set as 6 min. The thickness of the SnCu@Al thin film was ~ 20 nm, determined with a Surface Profiler (Bruker DektakXT) after depositing on silicon wafers with the same experimental condition as mentioned above.

Electrode preparation: The NVP electrode was prepared by casting the slurry of NVP/super P/PVDF in N-methyl-2-pyrrolidine with a mass ratio of 8/1/1 on Al foil. The mixture was vacuum-dried at 80 °C for 10 h. The mass loading of NVP is about 4.5 mg cm^{-2}. The diameter of the cathode electrode was 12 mm.

Characterization: The surface morphologies of Sn@Al, SnNi@Al, SnCu@Al, Na@Al, and Na@SnCu@Al were examined with field emission scanning electron microscopy (Tescan MAIA3) and transmission electron microscopy (JEOL JEM-2100F). The crystalline structure was characterized by X-ray diffraction using Cu Kα radiation (Rigaku SmartLab 9 kW Advanced). The surface morphology characterization of plated Na was conducted with atomic force microscopy (Bruker, Santa Barbara, CA) inside an Ar-filled glovebox. An area of 20 μm × 20 μm was randomly selected to detect the morphology and calculate the roughness. The surface chemical information of plated Na after 30 cycles was studied with X-ray photoelectron spectroscopy (XPS) (PHI 5000 VersaProbe II). All XPS samples were prepared within an argon-filled glove box. The peaks were calibrated by the C 1s peak at 284.8 eV. XPS shows an atomic ratio of 1.27 for Cu to Sn, close to the resultant formation of Cu_6Sn_5 from XRD pattern.

Electrochemical tests: All cells (CR2032-type coin cells) were assembled in an Ar-filled glove box (H_2O< 0.1 ppm; O_2< 0.1 ppm). 1 M $NaPF_6$ in diglyme was used as the electrolyte and the amount of electrolyte was fixed at 80 μL. One piece of f GF/D and one piece of Celgard 2325 were applied as separators. Na/Al cells were assembled with Na metal pairing with Al, Sn@Al, SnNi@Al, and SnCu@Al. The diameters of the different substrates and Na foils are 19 mm and 12 mm, respectively. To clean the surface and avoid the side reactions, the cells were activated at 50 μA cm^{-2} from 0.01 to 0.2 V versus Na/Na^+ for 5 cycles before cycling. The cut-off voltage was set to 0.2 V. For symmetric cells, 6.25 mAh cm^{-2} of Na was firstly deposited on the Al and SnCu@Al at 0.5 mA cm^{-2} to achieve the Na@Al and Na@SnCu@Al electroldes. For tafel plots, LSV tests were conducted with symmetric cells with a sweep rate of 1 mV s^{-2}. EIS was performed with a potential amplitude of 5 mV over a frequency range from 100 kHz to 10 mHz. Both LSV and EIS measurements were carried out with a Biologic VMP-3. For Na/NVP full cells, Na metal was pre-deposited on Al and SnCu@Al foils and the N/P ratio was controlled to 1. The anode-free NVP full cells were fabricated with Al and SnCu@Al foils and NVP cathode. All the full cells were tested with voltage ranging from 2.5 V to 3.8 V.

Supplementary Figures

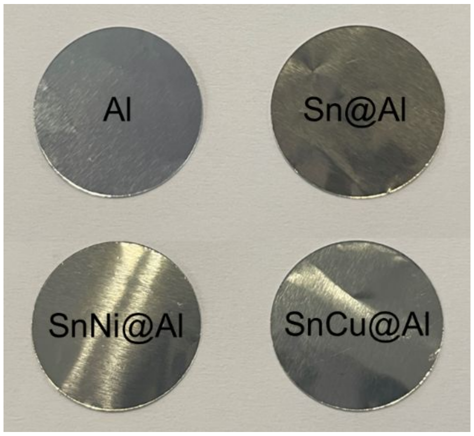

Figure S1. Optical images of (a) bare Al, (b) Sn@Al, (c) SnNi@Al, and (d) SnCu@Al.

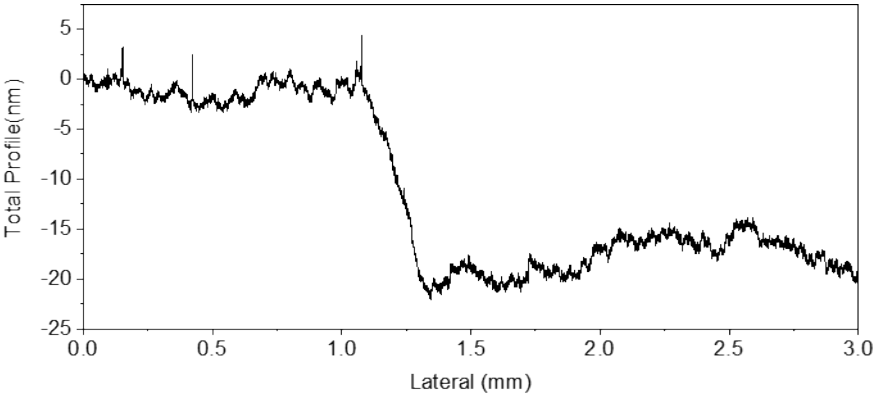

Figure S2. The thickness of the Sn-Cu coated layer was determined with the Surface Profiler.

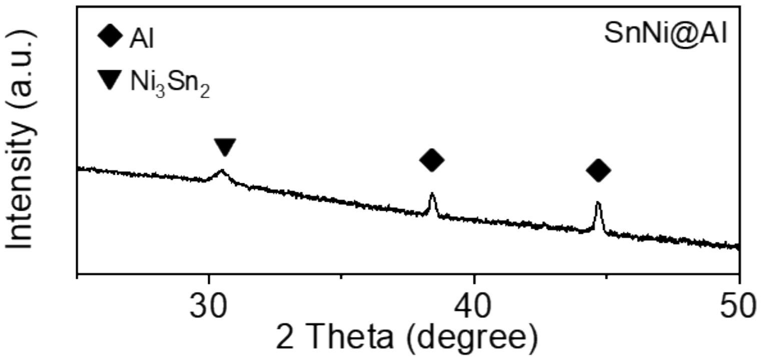

Figure S3. XRD patterns of SnNi@Al.

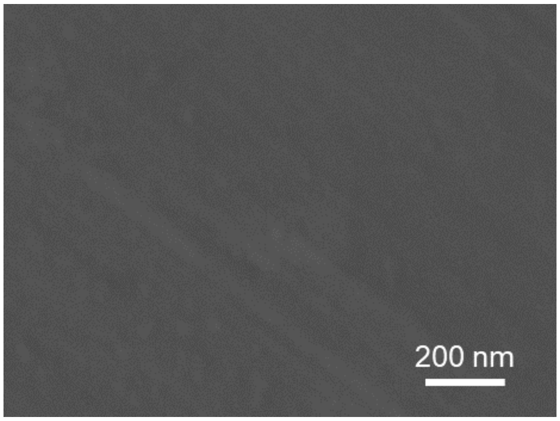

Figure S4. SEM image of bare Al.

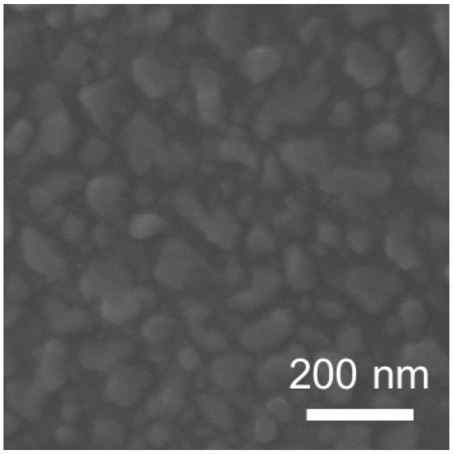

Figure S5. SEM image of SnNi@Al.

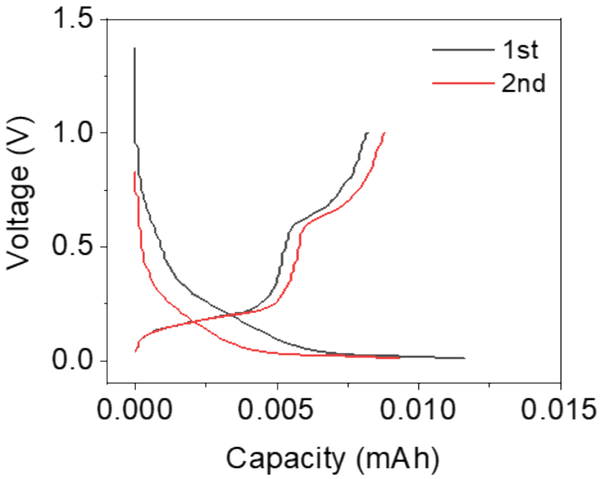

Figure S6. Galvanostatic discharge/charge curves of SnCu@Al during activation.

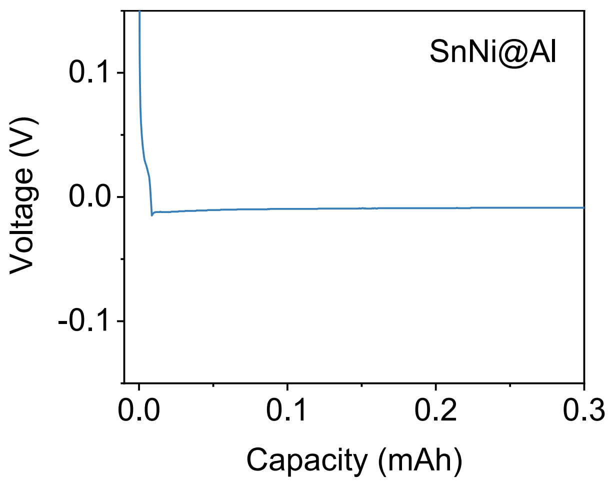

Figure S7. Nucleation overpotentials of Na plating on SnNi@Al at 0.2 mA cm^{-2}.

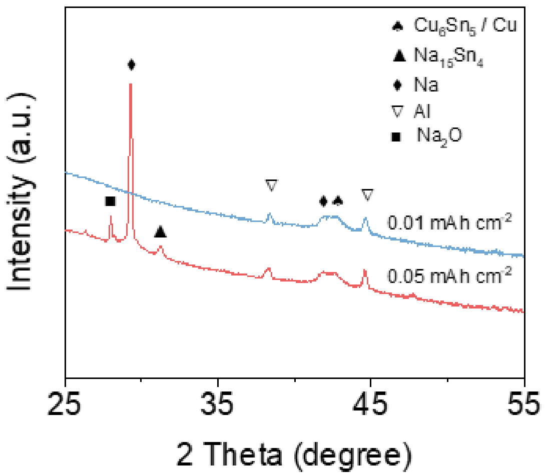

Figure S8. XRD patterns for the SnCu@Al electrode with a capacity of 0.01 and 0.05 mAh cm^{-2} Na plated at a small current density of 0.05 mA.

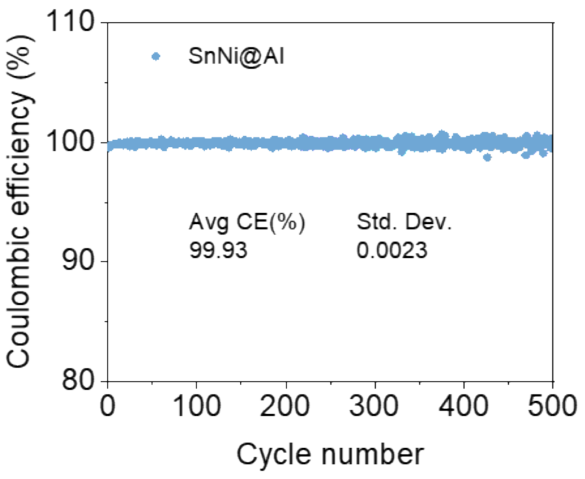

Figure S9. CE of Na/Al half cells using the SnNi@Al electrode at 1 mA cm^{-2} and 1 mAh cm^{-2}.

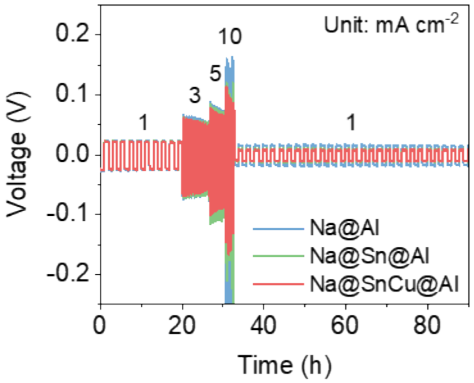

Figure S10. Rate performance of a) the Na@Al, Na@Sn@Al, and Na@SnCu@Al symmetric cells from 1 mA cm^{-2} to 10 mA cm^{-2}.

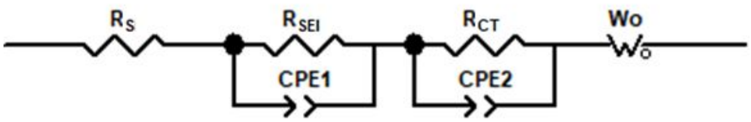

Figure S11. Circuit diagram of symmetric cells for the fitting of the Nyquist plots. Here, Rs represents the ion transport in electrolyte. R_{SEI} represents interfacial resistance across the SEI while R_{CT} means the charge transfer on the interface. CPE1 and CPE2 refer to the constant phase elements.

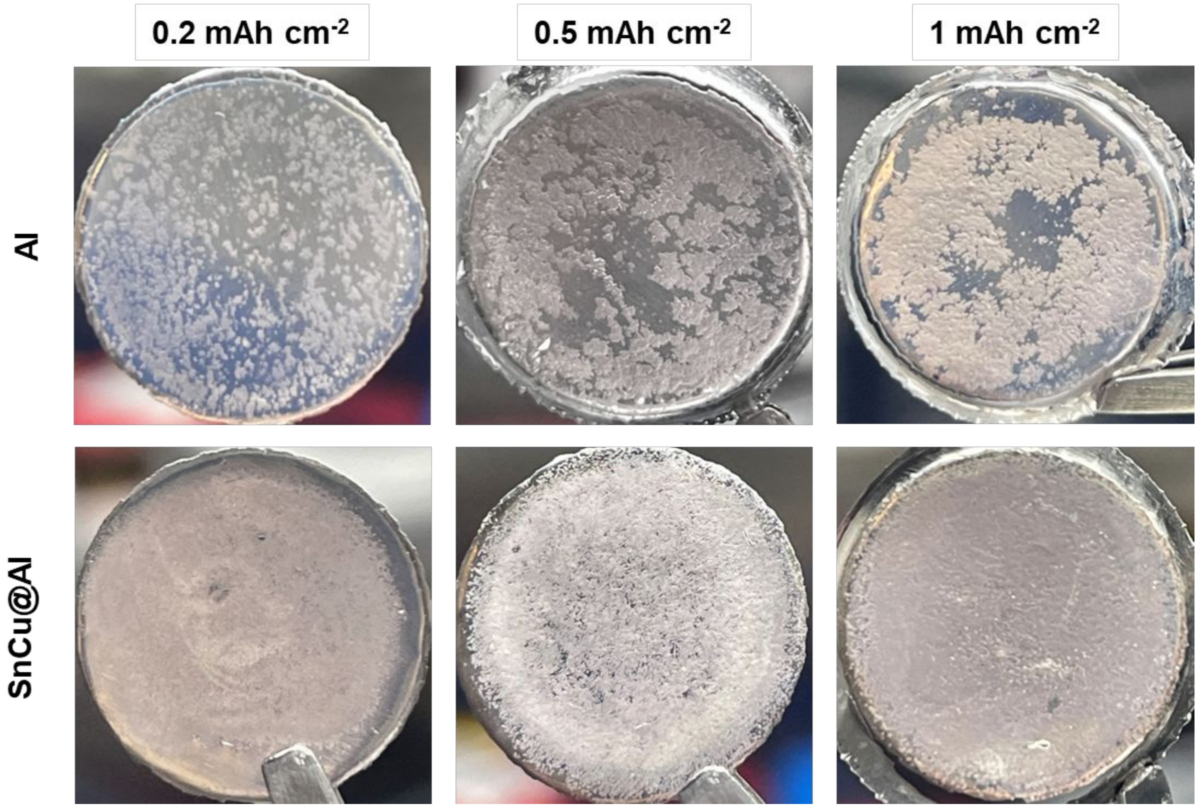

Figure S12. Optical images of Na plating on Al and SnCu@Al with the capacity of 0.2, 0.5, 1 mAh cm^{-2}.

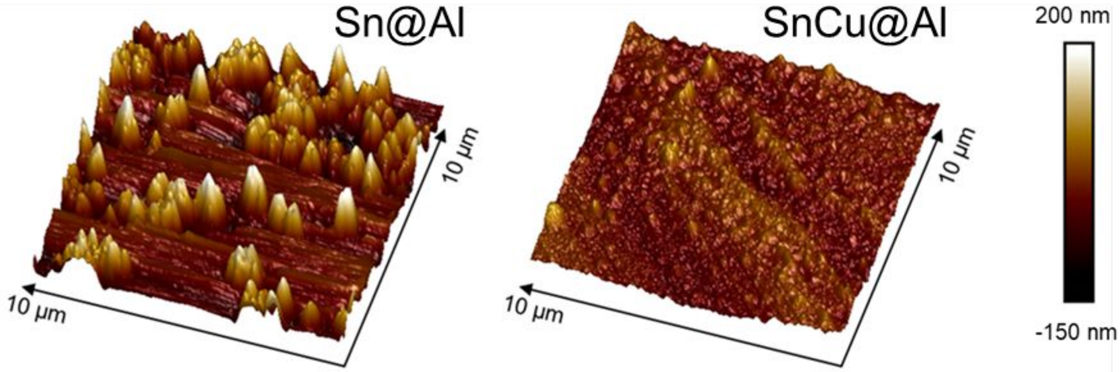

Figure S13. AFM 3D topographic mapping results of Sn@Al and SnCu@Al after 100 cycles plating and stripping at 2 mA cm^{-2} and 1 mAh cm^{-2}.

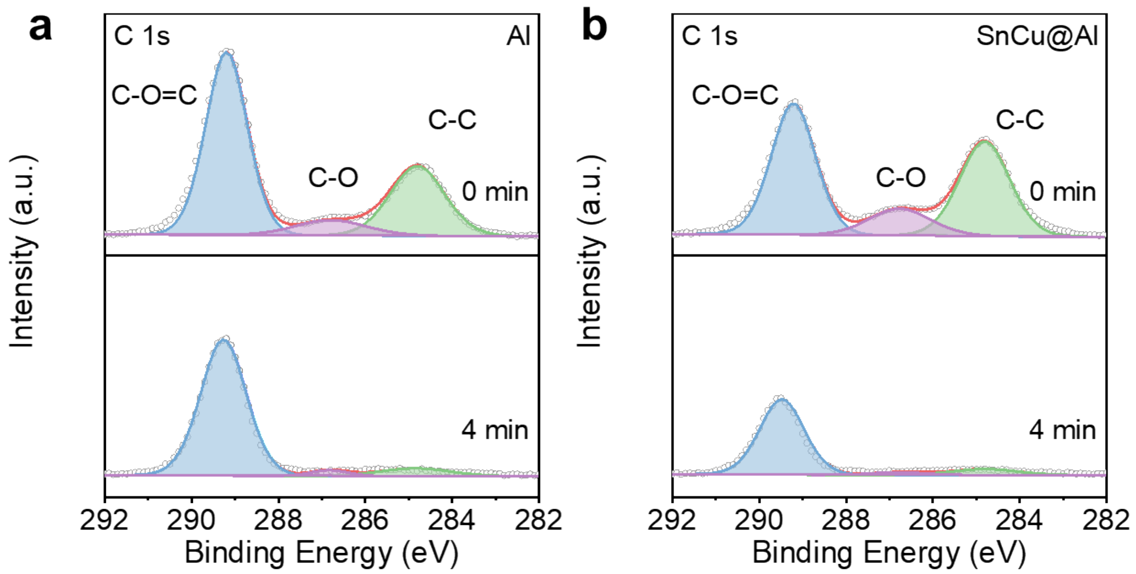

Figure S14. Depth-profiling C 1s XPS spectrum of Na plating on (a) Al and (b) SnCu@Al electrodes after 30 cycles at 2 mA cm^{-2} and 2 mAh cm^{-2}.

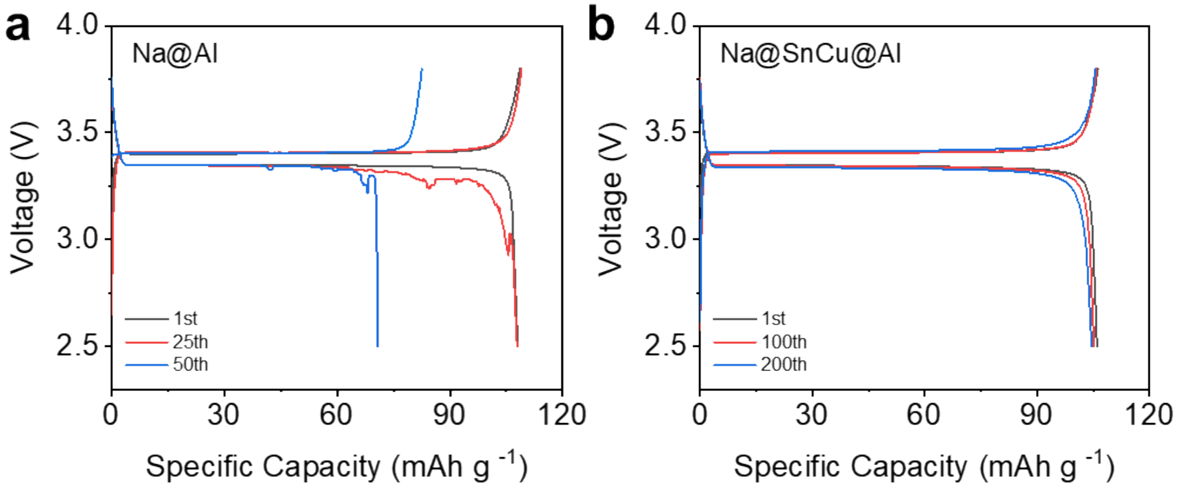

Figure S15. Typical charge/discharge curves of (a) Na@Al/NVP and (b) Na@SnCu@Al/NVP full cells at 1 C.

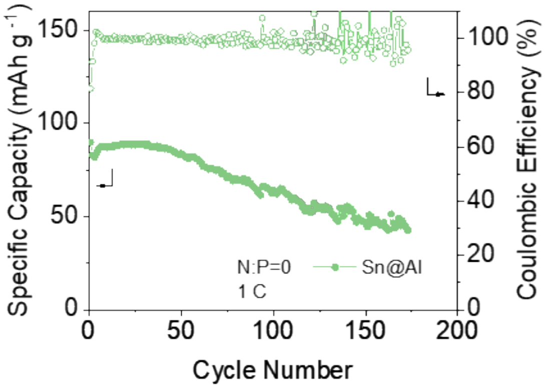

Figure S16. Cycling performance of Sn@Al/NVP anode-free full cells at 1 C.

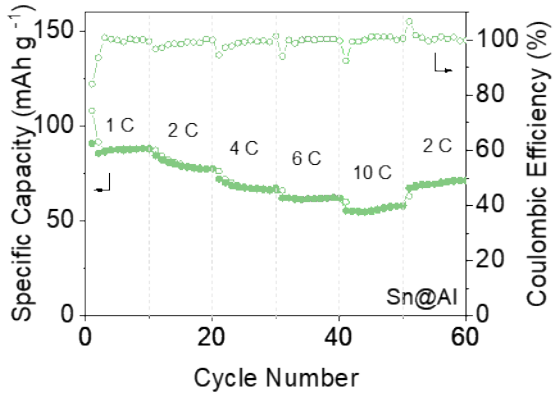

Figure S17. Rate performance of the Sn@Al/NVP cell cycling at increasing rates from 1 to 10 C.

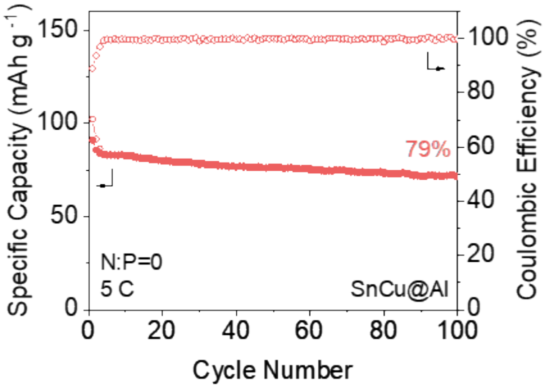

Figure S18. Cycling performance of the anode-free SnCu@Al/NVP cell at 5 C.
